# Secular Trends in Prevalence of Overweight and Obesity from 2006 to 2009 in Urban Asian Indian Adolescents Aged 14-17 Years

**DOI:** 10.1371/journal.pone.0017221

**Published:** 2011-02-23

**Authors:** Deepak Kumar Gupta, Priyali Shah, Anoop Misra, Swati Bharadwaj, Seema Gulati, Nidhi Gupta, Rekha Sharma, Ravindra M. Pandey, Kashish Goel

**Affiliations:** 1 National Diabetes, Obesity and Cholesterol Diseases Foundation (N-DOC), SDA, New Delhi, India; 2 Diabetes Foundation (India), SDA, New Delhi, India; 3 Department of Diabetes and Metabolic Diseases, Fortis Flt. Lt. Rajan Dhall Hospital, New Delhi, India; 4 Department of Biostatistics, All India Institute of Medical Sciences, New Delhi, India; 5 Department of Pediatrics, Children Hospital of Michigan, Detroit, Michigan, United States of America; 6 Department of Internal Medicine, Wayne State University, Detroit, Michigan, United States of America; The University of Queensland, Australia

## Abstract

The present study examines the secular trends in prevalence of overweight and obesity among urban Asian Indian adolescents in New Delhi (North India). The data were derived from cross-sectional sampling of children, 3493 in year 2006 and 4908 in year 2009, aged 14–17 years studying in privately-funded and government-funded schools. Age, gender and Asian Indian-specific cut offs of body mass index (BMI) were used to define overweight and obesity. The prevalence of obesity increased significantly from 9.8% in 2006 to 11.7% in 2009 (*p*<0.01), whereas underweight decreased from 11.3% to 3.9% (*p*<0.001). There was a significantly higher risk of being overweight (OR 1.28; 95% CI, 1.15–1.42) and obese (OR 1.44; 95% CI, 1.24–1.66) in year 2009 than 2006, after adjusting for age, gender and type of school. Males and privately-funded school children had significantly higher increase in prevalence and risk of being overweight and obese over the three years. In conclusion, this study showed an increasing trend in prevalence of overweight and obesity in urban Asian Indian adolescents. More specifically, the study showed the association of this increasing trend of overweight and obesity prevalence with male gender and high socio-economic status, calling for an urgent need for immediate and targeted preventive measures.

## Introduction

The epidemic of childhood obesity is a substantial health burden worldwide [1], [2], [3] and its impact is being observed in developing countries as well [4], [5]. Recent studies from western populations have shown a plateau in the prevalence of both adult and childhood obesity during the last decade [6], [7], [8]. However, the problem is of a larger magnitude in developing countries like India where a significant proportion of the population belongs to younger age group [9]. Rising prevalence of obesity in India may be attributed to various factors, like sedentary life-style, unhealthy food habits, cultural practices and increasing affluence of middle class population [10], [11], [12], [13]. Further, obesity is associated with multiple co-morbidities such as type 2 diabetes mellitus, dyslipidemia, polycystic ovarian disease, hypertension, and the metabolic syndrome, which are increasingly becoming common among children and urban adolescents [2], [4], [11], [12], [13]. Most importantly, childhood obesity has been associated with higher risk of morbidity and mortality in adult life [14].

While under-nutrition in children has been the major public health concern in India over the past several decades [15], little attention has been paid to childhood overweight and obesity until recently. The emerging evidence suggests an increase in over-nutrition status among children as well as adults [4], [16]. The National Family Health Survey (NFHS-3) 2005–2006 data showed that combined prevalence of obesity (body mass index ≥25 kg/m^2^) was 9.3% and 12.6% among men and women aged 15–49 years, respectively [17]. Previously, our group and others have reported prevalence of overweight in the range of 22–25% and obesity prevalence of 2–6% in children and young adolescents of Delhi [4], [18], [19].

However, limited literature is available on the trends of prevalence of childhood obesity in India. While one time cross-sectional studies do provide a good overview and snapshot of burden of obesity, these studies do not give detailed insight into the patterns and dynamics of this burgeoning problem. Studying trends of changes in prevalence of overweight and obesity becomes important as it allows the researchers and policy makers to design specific and targeted programs aimed at checking the problem of obesity. The aim of our study was to determine the secular trends in health status of urban Asian Indian adolescents (14–17 years age) and see if a trend similar to developed countries also exists in India.

## Materials and Methods

### Study populations

This study is based on data from a school-based program, named **M**edical Education for children/**A**dolescents for **R**ealistic prevention of obesity and diabetes and for healthy a**G**eing (*MARG-* Hindi for Path), being conducted in New Delhi [20]. The MARG program is the first large scale community intervention project in South Asia which focuses on the primary prevention of non-communicable diseases in children. In Phase 1 (year 2006–2007) anthropometric measurements were undertaken in 3493 urban adolescent children (aged 14–17 years) from government-funded (government funded or aided catering to children from low or lower middle income group families) and privately-funded (run by private groups catering to children from affluent families) schools of Delhi [4]. In the second phase, conducted in year 2008–2009, similar measurements were done in 4908 children (aged 14–17 years) from the government-funded and privately-funded schools, different from phase 1, of Delhi. In the present analysis, we present the secular trends in prevalence of overweight and obesity over the period of 3 years.

### Enrollment of Subjects

A list containing the names of all senior secondary government (or public) and non-government (or private) schools located in New Delhi was prepared. Five government schools and five private schools, different in two phases, were randomly selected and enrolled for the study. Generally, age 14 and 17 correspond to Standard IX and XII, respectively in the Indian Education system. Each standard is further divided into sections depending on the number of students. Two to three sections were randomly selected in all standards on the basis of number of students. All children of the selected sections were enrolled for the study and measured for height and weight. There was a 100% response from the children of selected schools in this study. Only children without history of any active disease or significant past medical history were included in the study.

### Ethics Statement

The study was ethically approved by the Institutional Review Board of Diabetes Foundation (India) and informed written consent was obtained from parents/guardians of school children after seeking permission from the principals to conduct the study in their schools.

### Measurements

The body measurements of weight and height were carried out by a trained physician and nutritionist, as described previously [21], [22] and were used to calculate the body mass index (BMI). Age and gender specific cut-offs for overweight and obesity were recently developed by our group for Asian Indian adolescents [23] using the LMS method defined by Cole *et al* [24] for IOTF cut-offs. In short, smoothed 5^th^, 10^th^, 25^th^, 50^th^, 75^th^, 85^th^, 90^th^, and 95^th^ percentiles for the various anthropometric parameters including BMI were derived using the Least Mean Squares (LMS) method for constructing normalized growth standards. In this study, underweight was defined as the 5^th^ percentile of the same reference population, as no defined cut-offs exist for Asian Indian children. Overweight and obesity were defined as the 85^th^ and 95^th^ percentile of this reference population, respectively. Using age, gender and ethnicity specific cut-offs is important as standard cutoffs of BMI used for adult or adolescent western population may not be applicable in studying the problem of childhood obesity in Asian Indians children [25], [26].

### Statistical Analysis

Data were managed on Excel spreadsheet with double-checking for errors. The differences between the two groups (2006 and 2009) were calculated using Chi-square test for categorical variables and Student's t-test for continuous variables. Multivariate Logistic Regression models were applied to assess the risk of being overweight and obese in the year 2009 as compared to 2006, after adjusting for age, gender and type of school in different groups. A p value<0.05 was considered significant. Data analysis was done on SPSS (version15; SPSS Inc., Chicago, IL, USA) software.

## Results

Study population consisted of 3493 (year 2006) and 4908 (year 2009) 14–17 year old children from privately funded and government funded schools of Delhi, India. Gender distribution did not show any significant difference (males: 63.7% in 2006 & 62.9% in 2009; *p* = 0.441), while significantly more children were enrolled from privately funded schools in 2009 as compared to 2006 (73.1% *vs.* 58.1%; *p*<0.001). Mean age of study populations was 15.09±1.04 years in 2006 and 15.25±1.04 years in 2009 (*p*<0.001). The overall mean BMI increased significantly from 19.3±4.3 kg/m^2^ to 20.2±4.2 kg/m^2^ (*p*<0.001). On stratification, mean BMI value increased significantly in males and both, privately-funded and government-funded schools (Table 1).

**Table 1:** Changes in BMI (Kg/m^2^) Values from 2006 to 2009.

Category	2006	2009	p Value
**Males**	18.7±4.2	20.0±4.0	0.006
**Females**	20.3±4.3	20.5±4.0	0.141
**Privately-funded Schools**	19.8±4.3	21.3±4.1	0.001
**Government-funded Schools**	17.9±3.7	18.7±3.4	0.005
**Total**	19.3±4.3	20.2±4.2	0.000

**Figure 1:**
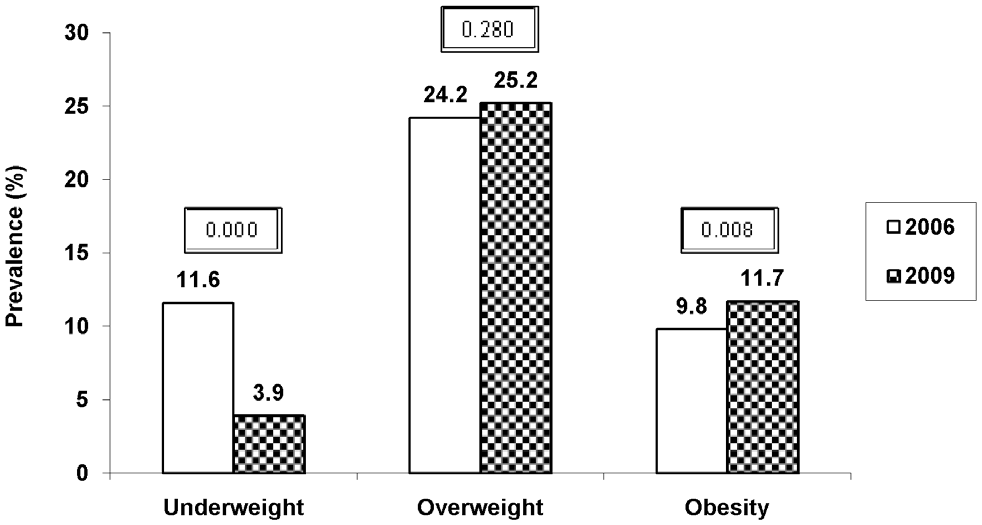
Secular Trends in prevalence of underweight, overweight and obesity from 2006 to 2009. Caption: Year 2006 (white bars) and 2009 (black bars) included 3493 and 4908 urban Asian Indian adolescents aged 14–17 years, respectively; *p* value<0.05 was considered significant; Underweight, overweight and obesity were defined as 5th, 85th and 95th percentiles of age and gender specific cut-offs of body mass index in Asian Indians, respectively [23].

Prevalence of overweight increased non-significantly from 24.2% in 2006 to 25.2% in 2009 (*p* = 0.280) while over same duration, obesity prevalence increased significantly from 9.8% in 2006 to 11.7% in 2009 (*p* = 0.008) (Figure 1). After adjusting for age, gender and type of school, there was a significantly higher risk of being overweight (OR 1.28; 95% CI, 1.15 – 1.42; *p*<0.001) and obese (OR 1.44; 95% CI, 1.24 – 1.66; *p*<0.001) in year 2009 than 2006 (Table 2).

**Table 2:** Multivariate logistic regression for the change in prevalence of overweight and obesity from 2006 to 2009.

Category	2006*	2009 OR (95% CI)			*p* value		
		Underweight	Overweight	Obesity	Underweight	Overweight	Obesity
**Males**	1	0.21 (0.16–0.26)	1.45 (1.27 – 1.66)	1.63 (1.35 – 1.97)	<0.001	<0.001	<0.001
**Females**	1	0.35 (0.26–0.47)	1.03 (.86 – 1.22)	1.18 (0.94 – 1.48)	<0.001	0.78	0.16
**Privately-funded** **schools**	1	0.19 (0.14–0.26)	1.31 (1.17 – 1.47)	1.55 (1.33 – 1.81)	<0.001	<0.001	<0.001
**Government-funded** **schools**	1	0.28 (0.22–0.36)	1.14 (0.89 – 1.46)	0.93 (0.65 – 1.35)	<0.001	0.30	0.71
**Overall**	1	0.255 (0.21 – 0.31)	1.28 (1.15 – 1.42)	1.44 (1.24 – 1.66)	<0.001	<0.001	<0.001

* 1 is reference for all models; p value<0.001 considered significant; OR, Odds ratio; 95% CI, 95% Confidence Intervals; All models are adjusted for age, gender and type of school.

**Figure 2:**
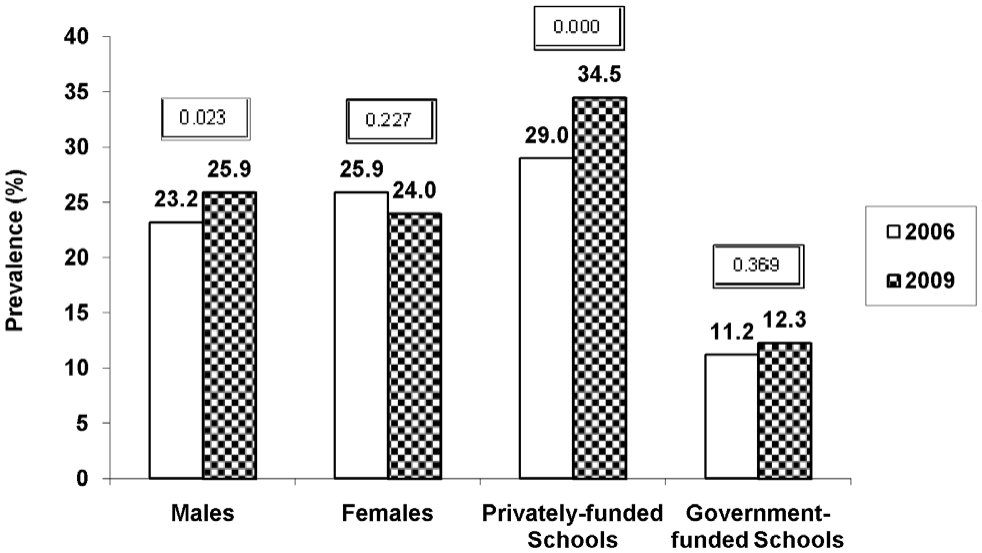
Secular trends in prevalence of overweight according to different gender and type of school from 2006 to 2009. Caption: Prevalence of overweight (in percentage, y-axis) in males, females, privately-funded and government-funded schools (x-axis) in year 2006 (white bars) and 2009 (black bars); *p* value<0.05 was considered significant; Overweight was defined as 85th percentile of age and gender specific cut-offs of body mass index in Asian Indians [23].

**Figure 3:**
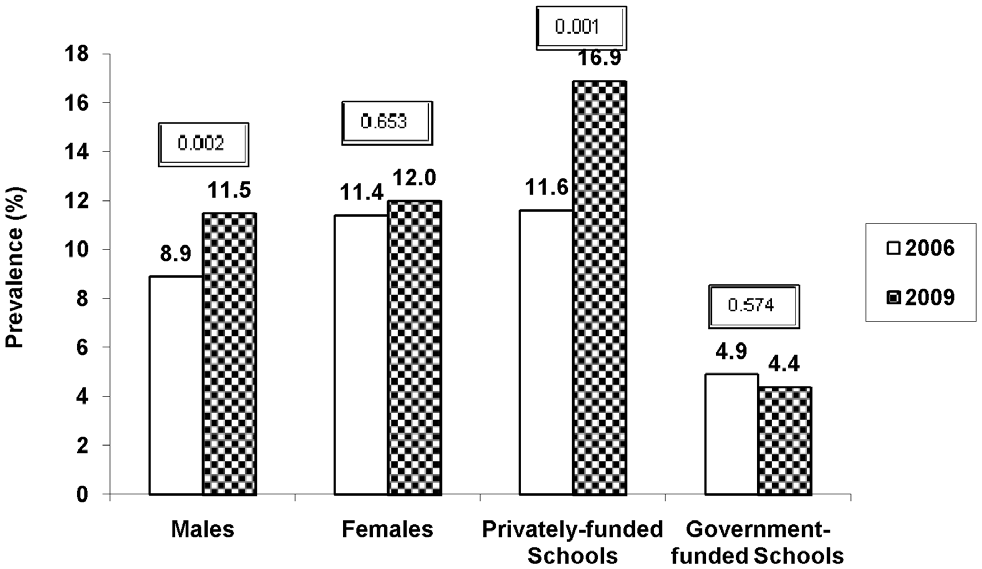
Secular trends in prevalence of obesity according to different gender and type of school from 2006 to 2009. Caption: Prevalence of Obesity (in percentage, y-axis) in males, females, privately-funded and government-funded schools (x-axis) in year 2006 (white bars) and 2009 (black bars); *p* value<0.05 was considered significant; Obesity was defined as 95th percentile of age and gender specific cut-offs of body mass index in Asian Indians [23].

Males showed a significant increase in prevalence of both overweight (23.2% to 25.9%; p = 0.023) and obesity (8.9% to 11.5%; *p* = 0.002) (Figure 2 & 3). In females, the prevalence of overweight decreased over 3 years (25.9% to 24.0%; *p* = 0.227) and though the prevalence of obesity in females was higher than males in both phases, there was only a small non-significant increase in its prevalence (11.4% to 12.0%; *p* = 0.653) (Figure 2 & 3). Over the three years, male gender was associated with a higher risk of being overweight (OR, 1.45; 95% CI, 1.27– 1.66; *p*<0.001) and obese (OR, 1.63; 95% CI, 1.35 – 1.97; p<0.001) than females in year 2009 than 2006, after adjusting for age and type of school (Table 2).

Privately-funded schools had a significant increase in prevalence of both overweight (29.0% to 34.5%; *p* = 0.000) and obesity (11.6% to 11.9%; *p* = 0.001) while government-funded schools did not show a significant change in prevalence of either overweight (11.2% to 11.3%; *p* = 0.369) or obesity (4.9% to 4.4%; *p* = 0.574) (Figure 2 & 3]. Children in the private schools were also at higher risk of being overweight (OR 1.31; 95% CI 1.17 –1.47; *p*<0.001) and obese (OR 1.55; CI 95% 1.33 – 1.81; *p*<0.001) than their government school counterparts, after adjusting for age and gender (Table 2).

No distinct pattern was observed in the prevalence trends across different age-groups (Data not shown). Of note, prevalence of underweight decreased significantly (11.6% to 3.9%; p = 0.000) from year 2006 to 2009 (Figure 1). The decrease was consistently significant among males and females, type of schools attended and all age-groups except 17 year old children (Data not shown).

## Discussion

This is the largest study on secular trends in the prevalence of childhood obesity in the Indian subcontinent. Our study demonstrated an increase in prevalence of both overweight and obesity and a decrease in prevalence of underweight in Asian Indian urban adolescents aged 14–17 years over a period of 3 years from year 2006 to 2009. Further, we report that this increase was significantly higher in males and children from affluent families.

Emerging problem of childhood obesity is of high importance in developing countries like India. India is going through concurrent transitions related to epidemiological, demographic and nutritional factors. Further, India is currently in the fourth phase of nutritional transition which is the shift of nutritional intake from basic to diet-related non-communicable diseases. These shifts are largely associated with behavioral changes in dietary profile and lifestyle and decreased indulgence in physical activity [27]. The transitions are more rapid in young individuals. This increasing trend of childhood overweight and obesity may further increase the enormous burden of type 2 diabetes and cardiovascular diseases in India [28], [29] and impact the economy of the nation and its growth.

Published literature on the prevalence of childhood obesity in India consists mainly of cross-sectional studies in different regions of the country, reporting its burden at a specified time. Studies from South India have reported an obesity prevalence of 3.6% in adolescents of age-group 13–18 years of Chennai in year 2002 [30] and 3.4% in children and adolescents of age-group 5–16 years of Mysore in year 2009 [31]. Several cross-sectional studies have been published from North India reporting the childhood obesity prevalence in the range of 3.6–7.0% [4], [18], [32], [33]. However, only one study, from Kerala (South India), has reported secular trend in the prevalence of childhood obesity [34]. These authors reported a significant increase in the prevalence of overweight and obesity from 4.94% and 1.26% in 2003 to 6.57% and 1.89% in 2005, respectively, in children aged 5–16 years. The increasing trend was noted in both sexes and privately-funded schools only. However, the investigators used CDC-defined cut-offs for determining the overweight and obesity prevalence. As the reference population for CDC cut-offs did not include Asian Indians, these cut-offs may not accurately represent the burden of childhood obesity in India. On the other hand, we used ethnic-specific cut-offs for our study population, which have been previously reported in adult Asian Indian populations also [21], [26], [35].

Most of the studies from India have reported the prevalence of childhood obesity using IOTF cut-offs, whose reference population did not include Asian Indians. We have previously reported that Asian Indians have a differential body composition as compared to white Caucasians and the universal cut-offs for overweight and obesity may not be applicable to this ethnic group [10], [12], [26]. Therefore, the use of IOTF cut-offs may have underestimated the prevalence of overweight and obesity in Asian Indian children and adolescents. To overcome these lacunae, we used the age and gender specific cut-offs for defining overweight and obesity in urban Asian Indian adolescents [23], developed using the same method as IOTF cut-offs.

Studies on adolescents in western population have shown that the prevalence of obesity has reached a plateau in the recent years [6], [7], [8]. The prevalence of overweight and obesity was 31.7% and 16.9% in 2007–2008 in North American children and adolescents aged 2–19 years, with no statistically significant trends noticed over the time periods of 1999–2000 until 2005–2006 [8]. A recent study from Switzerland also reported stabilization of trends in prevalence of childhood overweight and obesity from 1999 to 2005 [7]. This may imply that the developed countries are in the final stage of nutritional transition. However, this may not be the case in developing countries like India. Developing countries are still in the fourth stage on transition noticing an increase in the intake of fat and calorie dense foods leading to increased prevalence of obesity [29].

Another significant observation of this study was the independent association of male gender and high socio-economic status with the risk of being overweight and obese in childhood. Both these groups showed a significantly increased prevalence and higher risk of being overweight and obesity over the period of three years. Hormonal, cultural and social factors may account for the observed gender differences [36], especially in Indian context. Also, it has been seen that post-pubertal girls are more conscious towards their physical appearance and consequently may take active steps to control obesity as compared to boys [37]. Socio-economic status is another factor which has been linked to problem of overweight and obesity in many other studies [18], [30], [33], [38], [39], [40]. The assumption that type of school attended reflects the socio-economic status has been used and validated previously [41], [42]. There may be several explanations for this observation. Being financially sound may allow the children to indulge in practice of purchasing calorie dense fast foods and a life-style involving less of physical activity and more in-door activities like playing games on computer, watching television, etc. Also, the cultural beliefs in this region of the world like being overweight being considered as a marker of prosperity and good health may play a major role.

We also calculated trends in the prevalence of underweight in studied population. Historically, under-nutrition and underweight were considered to be mainly associated with childhood morbidity and mortality in India. However, with the increase in childhood obesity, an opposite trend is being noticed. Prevalence of underweight decreased significantly by 7.7% over the study period of 3 years. The results were consistently noted for all groups included in this study. This further supports our results and demonstrates a clear transition of Indian children from being underweight to becoming overweight and obese [28].

Our study has a few limitations. Firstly, the possibility of potential selection bias cannot be ruled out in a cross-sectional study spread across time and region. However, we tried to minimize the sampling bias. We compared individuals of same age group entering similar standards at two different points of time. We selected all students from randomly selected sections of a standard, with a 100% response rate from each section. Also, different schools were selected in two phases with the trend of private schools and government schools catering to high and low-socioeconomic status respectively remaining same over 3 years. Second, only urban children were included in study, with no representation from rural areas, which constitute quite a significant proportion of Indian population. Another limitation was that only BMI was used to determine overweight and obesity prevalence. Though recent studies have raised concerns on reliability of BMI as a measure of obesity in view of newer indices [43], [44], BMI still remains the most widely used measure in large population-based studies [6], [7], [8], [45], [46], [47], [48], [49] owing to its inherent simplicity to use, availability of population specific cut-offs and time tested reliability. Also, BMI has been shown to be comparable with other measures of obesity in risk assessment [50], [51]. Other sophisticated measures of obesity like percentage body fat could further add insights into these trends. However, using these measures require expensive instruments and necessary training to the field workers for their correct use. This limits their use in large scale studies, particularly in settings of developing countries like India, where financial and human resources are a significant limitation in clinical research. Abdominal obesity measures like waist circumference and waist-to-hip ratio, are simpler to employ and have been shown as an important and reliable index of obesity in various populations [52] including one study on Asian Indians [10]. However, no ethnicity-specific cut-offs exist for abdominal obesity in Asian Indian adolescents unlike western countries [53] **.** We are working on developing these ethnicity-specific cut-offs of abdominal obesity in a population-based study for use in future studies.

In spite of these limitations in mind, our study had several strengths. We included one of largest samples in urban Asian Indian adolescents with good representation from different group including age, gender and socio-economic status. In addition, we included age, gender and ethnicity specific BMI cut-offs to define childhood overweight and obesity, which may provide a more accurate estimate of their prevalence than previous studies.

### Conclusions

In conclusion, we report that the prevalence of obesity has increased significantly in the last 3 years in urban Asian Indian adolescents aged 14–17 years in North India. In addition, male gender and higher socio-economic status is associated with a significant risk of being both overweight and obese. Large scale nationwide campaigns targeted at these specific groups are required to check the growing epidemic of childhood obesity in developing countries. Countrywide awareness programs to spread healthy messages on good nutrition and good health for the prevention of obesity and its consequences need to be initiated. These shall not only promote good health, but shall also help in the prevention of non-communicable diseases as diabetes, heart problems, and other related diseases. On the long run, such programs shall act to reduce the burden on economic growth of the nation.
